# In vitro zinc supplementation alters synaptic deficits caused by autism spectrum disorder-associated *Shank2* point mutations in hippocampal neurons

**DOI:** 10.1186/s13041-021-00809-3

**Published:** 2021-06-24

**Authors:** Yukti Vyas, Yewon Jung, Kevin Lee, Craig C. Garner, Johanna M. Montgomery

**Affiliations:** 1 Department of Physiology and Centre for Brain Research, Faculty of Medical and Health Sciences, University of Auckland, Private Bag 92019, Auckland, New Zealand; 2 German Center for Neurodegenerative Diseases, Charité-Universitätsmedizin Berlin, Berlin, Germany

**Keywords:** Autism, Shank2, Zinc supplementation, Glutamatergic synapses

## Abstract

Autism Spectrum Disorders (ASDs) are neurodevelopmental disorders characterised by deficits in social interactions and repetitive behaviours. ASDs have a strong genetic basis with mutations involved in the development and function of neural circuitry. Shank proteins act as master regulators of excitatory glutamatergic synapses, and Shank mutations have been identified in people with ASD. Here, we have investigated the impact of ASD-associated Shank2 single nucleotide variants (SNVs) at the synaptic level, and the potential of in vitro zinc supplementation to prevent synaptic deficits. Dissociated rat hippocampal cultures expressing enhanced green fluorescent protein (EGFP) tagged Shank2-Wildtype (WT), and ASD-associated *Shank2* single nucleotide variants (SNVs: S557N, V717F, and L1722P), were cultured in the absence or presence of 10 μM zinc. In comparison to Shank2-WT, ASD-associated *Shank2* SNVs induced significant decreases in synaptic density and reduced the frequency of miniature excitatory postsynaptic currents. These structural and functional ASD-associated synaptic deficits were prevented by chronic zinc supplementation and further support zinc supplementation as a therapeutic target in ASD.

## Micro report

Shank2 is a postsynaptic density (PSD) protein that is located at excitatory glutamatergic synapses [5, 9]. Shank2 plays a major role in synapse development, where it interacts directly with other PSD proteins to regulate surface glutamate receptors and the actin cytoskeleton [5, 14]. S*HANK2* mutations have been identified in ASD patients, with *SHANK2*-single nucleotide variants (SNVs) detected in the SH3, PDZ, and proline-rich Shank2 domains [4, 11, 15]. Shank2 proteins have been shown to be zinc-sensitive: For example, Shank2 increases in hippocampal neurons supplemented with zinc [8], while chronic zinc deficiency induces loss of Shank2/3 and increased incidence of ASD-behaviours [8]. Supplementation of dietary zinc in Shank3^*−/−*^ mice also induces Shank2 recruitment to cortico-striatal synapses and reverses ASD-associated behaviours [6]. Transiently increasing synaptic zinc availability using clioquinol in vivo restored glutamatergic hippocampal synaptic transmission in *Shank2*^*−/−*^ mice through NMDA receptor activation [12]. However, zinc regulation of synaptic transmission in neurons expressing *Shank2* ASD-associated SNVs has not been investigated. Here, we explore the potential of in vitro zinc supplementation in preventing synaptic deficits in hippocampal neurons expressing ASD-associated *Shank2* SNVs.

Primary hippocampal dissociated cultures were prepared from male and female postnatal day zero (P0) Wistar rats as described previously [1, 2, 3, 7]. At days *in* vitro (DIV) 9, hippocampal cultures were transfected via calcium phosphate precipitation [7, 10, 13] with one of three ASD-associated SNVs occurring in highly conserved sites: (1) *Shank2*-S557N SNV (Serine to Asparagine amino acid change), identified at nucleotide position C70322303T in exon 13 of ASD patients and healthy controls, and is encoded in the Shank2 SH3 domain; (2) *Shank2*-V717F (Valine to Phenylalanine mutation), an SNV that occurs at nucleotide position C70026597A in exon 17, identified only in ASD patients, and is encoded in the Shank2 PDZ domain; and (3) *Shank2-*L1722P (Leucine to Proline mutation), presented at nucleotide position A69997007G in exon 25, identified only in ASD patients, and encoded in the Shank2 proline-rich region [11]. The *Shank2*-wildtype and *Shank2* SNV genes (S557N, V717F, and L1722P) were encoded into a 4.7 kb pEGFP-C1 vector including a neomycin resistance cassette, SV40 early promoter, neomycin/kanamycin resistance gene of Tn5, and polyadenylation signals from the Herpes simplex virus thymidine kinase (HSV TK) gene (B.D. Bioscience Clontech; gifted by Professor Craig C. Garner). Hippocampal cultures were treated with 10 µM ZnCl_2_ on DIV9 immediately after transfection for 5–7 days. A minimum of 3 independent culture preparations and transfections were examined for all data sets. Cultures were paraformaldehyde-fixed at DIV16 for immuno-staining for pre- and postsynaptic proteins using the following antibodies: anti-VGLuT1 (1:500, Neuromab N28/9, 75-066), rabbit anti-Homer (1:500, Santa Cruz Biotechnologies H-342), donkey anti-mouse IgG Alexa 594 (1:500, Molecular Probes A21203), and donkey anti-rabbit IgG Alexa 647 (1:500, Molecular Probes A31573). Z-stack images were acquired at 0.2 µm z-intervals using the Zeiss Axio Imager M2 Fluorescence Microscope at 63 × magnification with a 1.4NA oil immersion objective lens. Images were analysed using ImageJ Biophotonics (NIH, USA) to measure synaptic density defined by number of co-localised VGluT1 and Homer puncta per 10 µm length of dendrite Arons et al. [1, 2].

**Fig. 1:**
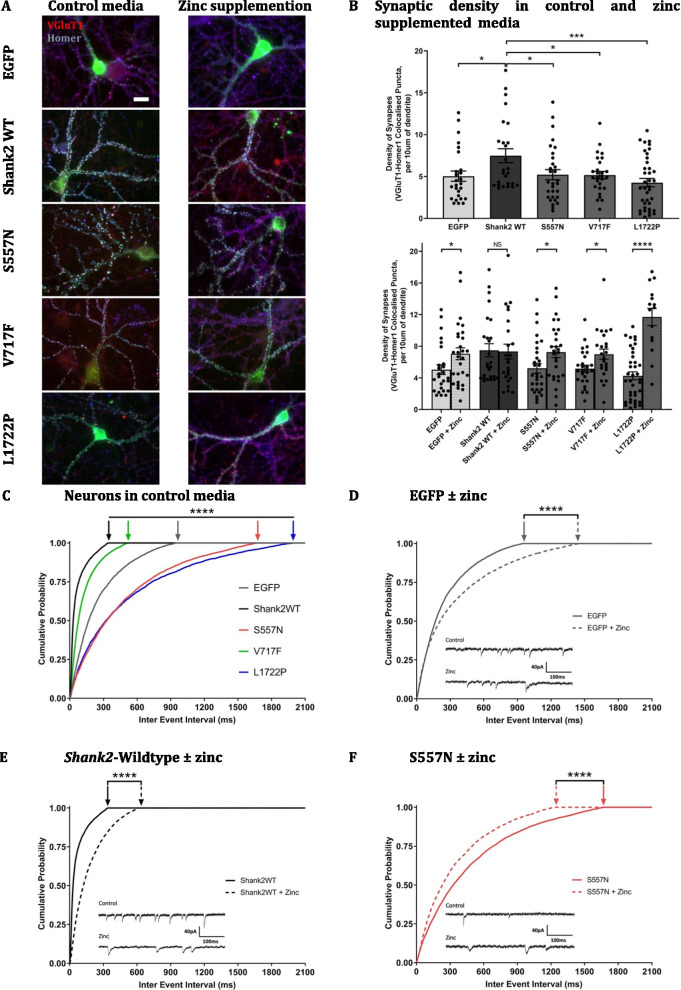

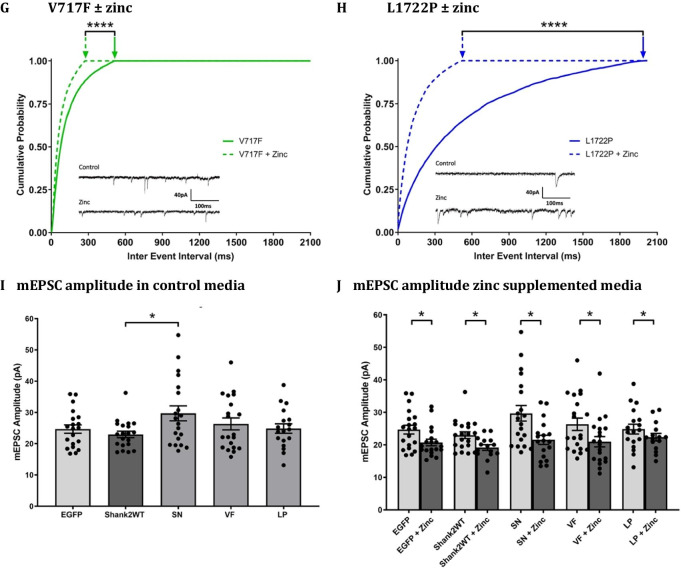
Influence of ASD-Shank2 SNVs on excitatory glutamatergic synapses. **A** Representative images of hippocampal neurons transfected with EGFP, *Shank2*-wildtype or ASD-associated *Shank2* SNVs, S557N, V717F or L1722P, cultured without (left) or with (right) 10 μM zinc supplementation from DIV 9 till DIV 16 (transfected neurons shown in green), and immuno-stained with VGluT1 (Alexa Fluor 594, shown in red) and Homer (Alexa Fluor 647, shown in blue). Scale bar is 20 μm. **B** Quantification of VGluT1 and Homer co-localised puncta density per 10 µm length of dendrite in hippocampal neurons transfected with EGFP, *Shank2*-wildtype or ASD-associated *Shank2* SNVs, S557N, V717F or L1722P (above) and in transfected hippocampal neurons supplemented with 10 µM zinc from days in vitro (DIV) 9 till DIV 16 (below). **C** Cumulative probability graph of miniature excitatory post-synaptic current (mEPSC) inter-event interval in neurons expressing EGFP, *Shank2*-wildtype or ASD-associated *Shank2* SNVs, S557N, V717F or L1722P. **D** Cumulative probability plot of mEPSC inter-event interval in control EGFP- and zinc treated EGFP-expressing hippocampal neurons. **E** Cumulative probability plot of mEPSC inter-event interval in control and zinc-treated *Shank2*-wildtype-expressing hippocampal neurons. (**F**–**H**) Cumulative probability plots of mEPSC inter-event intervals in S557N, V717F and L1722P-expressing neurons cultured in the presence of absence of zinc. Example mEPSC traces are shown for each Shank2 variant. **I**, **J** Bar graphs of mEPSC amplitudes from EGFP, Shank2WT, S557N, V717F, and L1722P-expressing neurons grown in control (**I**) and zinc supplemented (**J**) media. Data were statistically analysed using two-way ANOVA with Tukey’s multiple comparisons test. *NS*  not significant, *p < 0.05, **p < 0.01, ***p < 0.001, ****p < 0.0001.

All Shank2 SNVs showed similar cellular expression patterns in the dendritic and spine compartments in our cultured hippocampal neurons (Fig. 1A; intensity ratios dendrite/spine: Shank2 WT 0.671 ± 0.009; S557N 0.669 ± 0.016; V717F 0.677 ± 0.014; L1722P 0.685 ± 0.018). However, the L1722P SNV displayed a significantly expression ratio in the somatic/dendritic and somatic/spine expression ratios (intensity ratios soma/dendrite: Shank2 WT 1.549 ± 0.106; S557N 1.455 ± 0.067; V717F 1.604 ± 0.082; L1722P 2.770 ± 0.170, *p* < 0.0001; intensity ratios soma/spine: Shank2 WT 1.058 ± 0.078; S557N 0.983 ± 0.063; V717F 1.123 ± 0.063; L1722P 2.168 ± 0.183, *p* < 0.0001), reflecting its high localisation in the soma (Fig. 1A), and a potential difference in the subcellular trafficking of this SNV. Our immunocytochemical analysis showed Shank2-wildtype induced a significant increase in synaptic density compared to control EGFP-transfected neurons (*p*-value = 0.021; Fig. 1B; Table 1). In contrast, ASD-associated Shank2 SNVs failed to induce this significant increase in synapse density suggesting that these Shank2 SNVs are loss-of-function mutations (S557N: *p*-value = 0.029; V717F: *p*-value = 0.031; L1722P: *p*-value = 0.0003). This is consistent with data previously reported by Leblond et al., [11] showing decreased synaptic density in ASD-Shank2 transfected neurons compared to wildtype Shank2. Interestingly, the observed Shank2 SNV-induced reduction in synaptic density did not occur in the presence of chronic zinc supplementation, suggesting that chronic zinc supplementation can rescue SNV-associated deficits in their ability to promote synapse formation and/or maintenance (Fig. 1B, Table 1). This zinc prevention of synaptic density deficits was observed for all Shank2 SNVs, i.e. S557N, V717F, and L1722P, with the greatest increase in synapse density in L1722P expressing neurons (Fig. 1B, Table 1). Chronic zinc supplementation also increased synapse density in EGFP-transfected neurons expressing endogenous levels of Shank2, however, it did not further increase synapse density in neurons overexpressing the wildtype form of Shank2.

**Table 1:** Influence of zinc supplementation on glutamatergic synaptic density

Synapse density per 10 µm of dendrite ± standard error of the mean, N = number of neurons				
	Control	With Zn^2+^ Supplementation	*p*-value	Zinc-induced change in synapse density
EGFP	5.04 ± 0.61, N = 26	7.05 ± 0.83, N = 15	0.041	↑
Shank2-wildtype	7.49 ± 0.83, N = 26	7.34 ± 0.92, N = 24	0.83	–
Shank2-S557N	5.21 ± 0.61, N = 30	7.26 ± 0.69, N = 28	0.022	↑
Shank2-V717F	5.17 ± 0.44, N = 30	6.99 ± 0.65, N = 24	0.018	↑
Shank2-L1722P	4.26 ± 0.50, N = 28	11.70 ± 1.08, N = 15	< 0.0001	↑↑↑

To examine whether these structural synaptic changes translated to functional changes in synaptic transmission, whole-cell patch-clamp recordings were conducted between DIV 14–16 to measure miniaturised excitatory postsynaptic currents (mEPSCs). Cells were perfused with aCSF (in mM: 119 NaCl, 2.5 KCl, 1 Na_2_HPO_4_, 1.3 MgSO_4_, 26.2 NaHCO_3_, 11 D-( +)-glucose, 2.5 CaCl_2_) containing 1 µM Tetrodotoxin (TTX) and 100 µM picrotoxin. Internal solution consisted of (in mM): 120 K gluconate, 40 HEPES, 5 MgCl_2_, 2 Na_2_ATP, 0.3 NaGTP, pH 7.2 with KOH, 298 mOsm, and neurons were voltage-clamped at -65 mV. Recordings with series resistance variation greater than 20% were discarded. MiniAnalysis (Synaptosoft version 6.0.7) software was used to analyse the frequency and amplitude of spontaneous AMPAR-mediated mEPSCs. Zinc-dependent effects on mEPSC frequency were measured as alterations in the time intervals between events (inter-event interval, IEI; plotted as cumulative probability graphs, where a rightward-shifted IEI represents a decrease in mEPSC frequency and leftward-shifted IEI represents an increase in mEPSC frequency).

In control media (Fig. 1C), *Shank2*-wildtype expressing neurons displayed a significantly leftward-shifted IEI in comparison to EGFP-transfected neurons, indicating an increase in mEPSC frequency (Fig. 1D, E). Neurons expressing ASD-associated Shank2 SNVs were unable to cause this increase in mEPSC frequency in comparison to Shank2-wildtype (Fig. 1F–H). Varying extents of the loss of function were observed between the ASD-Shank2 SNVs, with *Shank*-V717F expressing neurons showing higher mEPSC frequencies than EGFP expressing neurons. In contrast, the S557N and L1722P mutations reduced mEPSC frequency even lower than EGFP neurons (expressing endogenous levels of Shank2; Fig. 1F, H). This may reflect domain-specific effects of each mutation on AMPA receptor function, localisation, and/or recruitment. Interestingly, in contrast to control Shank2 and EGFP-expressing neurons, zinc supplementation increased mEPSC frequency across all ASD-associated Shank2 SNVs expressing hippocampal neurons, demonstrating that in vitro zinc supplementation was able to prevent ASD SNV-associated deficits in glutamatergic synaptic transmission. The zinc-induced rescue was most profound in L1722P-expressing neurons, but was also significant in the V717F and S557N-expressing neurons (Fig. 1F–H). Therefore regardless of the domain in which the Shank2 SNV is present, chronic zinc can alter synaptic transmission partially or completely to control levels.

AMPAR mEPSC amplitudes were also observed to be altered differentially by Shank2 SNVs (Fig. 1I, J). Specifically, increased mEPSC amplitudes were only observed in neurons expressing Shank2-S557N, a mutation which may alter SHANK-GRIP interaction in the SH3 domain to prevent AMPAR internalisation. Chronic zinc supplementation significantly decreased mEPSC amplitude in S557N and V717F expressing neurons, as was also observed in control WT-Shank2 and EGFP-expressing neurons. Together our mEPSC data show chronic zinc primarily alters mEPSC frequency in neurons expressing Shank2-SNVs. This suggests the location of zinc action may differ in inducing changes in mEPSC frequency versus amplitude, e.g. potentially postsynaptic versus presynaptic effects, and that how zinc affects mEPSC amplitude is not altered by SNVs in these two sites. We did not observe a significant decrease in mEPSC amplitude in L1722P-expressing neurons; this is likely due to the lower initial amplitude of these currents, but could also reflect this SNV occurs in the Shank2 proline-rich region which is not involved with Shank protein interaction with glutamatergic receptors.

Together our imaging and electrophysiological data reveal that ASD-associated Shank2 SNVs cause structural and functional deficits at excitatory glutamatergic hippocampal synapses. Furthermore, chronic in vitro zinc supplementation induces long-lasting forms of structural and functional plasticity that in the presence of Shank2 SNVs can prevent or lessen the ASD-associated synaptic deficits. The Shank2 variants examined can exhibit differences in the extent they contribute to the synaptic aetiology of ASD*.* However, although the Shank2-SNVs investigated here occur in different domains of Shank2 thereby having differential effects of synapses and overall ASD aetiology, our data show that neurons expressing these Shank2 SNVs retain their zinc responsiveness. Our imaging data support both a pre- and a postsynaptic effect of zinc supplementation. As Shank3 has a trans-synaptic strengthening effect on both the pre- and postsynapse [2], as well as playing a key role in a zinc-sensitive signalling pathway at glutamatergic synapses [6], we predict zinc supplementation activates and recruits Shank3 to synapses and promotes AMPAR recruitment as well as act trans-synaptically to enhance presynaptic function in Shank2 SNV-expressing hippocampal neurons. These data indicate that altering the neuronal zinc micro-environment can prevent synaptic deficits caused by ASD-associated genetic alterations, and further support zinc supplementation as a therapeutic target in ASD.

## Data Availability

All data generated or analysed during this study are included in this published article.
